# Periodontitis during pregnancy: The effect on the gut microbiome and intestinal inflammation

**DOI:** 10.1002/jper.70132

**Published:** 2026-04-14

**Authors:** Richard Bright, Matthew G. Macowan, Keyuan Tian, Tracy Fitzsimmons, Rebecca L. Wilson, Claire T. Roberts, Claus T. Christophersen, Peter M. Bartold, Stephen P. Kidd, Peter S. Zilm

**Affiliations:** ^1^ College of Medicine and Public Health Flinders University Bedford Park South Australia Australia; ^2^ School of Biological Sciences University of Adelaide Adelaide South Australia Australia; ^3^ Adelaide Dental School University of Adelaide Adelaide South Australia Australia; ^4^ Robinson Research Institute University of Adelaide Adelaide South Australia Australia; ^5^ School of Medical and Health Sciences Edith Cowan University Perth Western Australia Australia

**Keywords:** adverse pregnancy outcomes, gut microbiome, inflammation, microbiota dysbiosis, periodontitis

## Abstract

**Background:**

Periodontitis has been epidemiologically associated with adverse pregnancy outcomes, but causality remains difficult to establish in humans due to confounding factors. This study uses a controlled murine model to examine the effects of experimentally induced periodontitis on the composition of the gut microbiota and gastrointestinal inflammation during pregnancy.

**Methods:**

Periodontitis was induced in pregnant BALB/c mice via oral inoculation with *Porphyromonas gingivalis* and *Fusobacterium nucleatum* before conception (*n* = 20 per group). Pregnancy outcomes, gut histology, systemic inflammatory markers, and microbiome composition, assessed by 16S rRNA sequencing, were evaluated at gestational Day 18.

**Results:**

Periodontitis was confirmed by significant alveolar bone loss. While fetal and placental weights were modestly increased in periodontitis‐positive mice, there were no changes in implantation rates or placental efficiency. Systemic inflammatory markers, including C‐reactive protein and interleukin‐33, were reduced, suggesting pregnancy‐specific immunomodulation. Histological analysis revealed significant inflammation in the jejunum and colon of periodontitis‐exposed mice. Despite this, alpha and beta diversity metrics of the gut microbiota remained essentially unchanged. Taxonomic shifts were observed at the genus level, with reductions in protective taxa, such as *Akkermansia muciniphila* and increases in potentially pro‐inflammatory genera, like *Desulfovibrio*.

**Conclusions:**

Periodontitis during pregnancy alters gut microbial composition and increases gastrointestinal inflammation without overtly impairing pregnancy outcomes in mice. These findings suggest an association between oral inflammation, intestinal inflammatory changes, and systemic inflammatory modulation during pregnancy. Further studies are warranted to explore long‐term maternal and offspring consequences and their relevance to human pregnancy.

**Plain language summary:**

This study explored how periodontitis during pregnancy can influence the gut and immune system. Periodontitis is already associated with poor pregnancy outcomes, but establishing cause and effect in humans is difficult. To investigate this, the researchers used a controlled mouse model. We induced periodontitis in pregnant mice and examined its impact on the gut microbiome, intestinal health, and immune responses. The results revealed that periodontitis does not stay confined to the mouth; it disrupts gut bacterial balance, causes gut inflammation, and modifies immune pathways. Notably, these effects occurred during pregnancy, a time when the immune system is already adapting. The findings suggest that oral infections during pregnancy can have widespread effects, impacting gut health and immune regulation. This may help explain the link between periodontitis and human pregnancy complications. Overall, the study underscores the importance of oral health during pregnancy and supports the idea that treating periodontitis might also safeguard gut and immune health, leading to better outcomes for both mothers and their babies.

## 1 INTRODUCTION

A substantial body of research links oral bacteria, particularly those associated with periodontitis, to systemic diseases as well as adverse pregnancy outcomes (APOs).^1^ These APOs comprise complications such as fetal growth restriction, preterm birth, low birth weight, miscarriage, and stillbirth, each of which poses significant risks to neonatal survival and long‐term developmental health. Current studies are unable to isolate the effects of periodontitis alone from other variables, such as diet, smoking status, weight, lifestyle, and many other factors that can impact pregnancy outcomes and therefore published literature is equivocal.^2^, ^3^, ^4^ During periodontitis, anaerobic, Gram‐negative species predominate in the subgingival regions of the mouth, particularly in periodontal pockets, where they form complex biofilms.^5^ The diffusion of biologically active products from developing subgingival biofilms into the gingival crevicular fluid and surrounding gingival connective tissue triggers the characteristic inflammation associated with periodontitis.^6^ *Fusobacterium nucleatum (F. nucleatum)* is an oral commensal that increases in abundance by approximately 1000‐fold during periodontitis.^7^ It co‐aggregates with many other oral bacteria,^8^ and can reduce the redox level of the periodontal pocket by producing NADH oxidase and peroxidase activity, creating an anaerobic environment.^9^, ^10^ *Porphyromonas gingivalis (P. gingivalis)* is another Gram‐negative, strict anaerobe and is one of the primary bacteria associated with periodontitis, along with *Treponema denticola* and *Tannerella forsythia*.^11^ It produces proteolytic gingipains that destroy and detach the periodontal ligament, leading to chronic inflammation.^12^, ^13^ Chronic inflammation stimulates osteoclast activity, leading to alveolar bone resorption.^14^ Eventually, this can lead to tooth loss and gingival bleeding, which can serve as portals for bacteria to enter the bloodstream.

Hormonal changes during pregnancy result in women being more susceptible to periodontal disease.^15^ Not only does the resultant gingival bleeding allow bacteria access to the bloodstream, but increases in estrogen and progesterone lead to heightened vascular permeability of the periodontium, further enabling hematogenous translocation to the fetoplacental unit.^15^ There are currently 2 prevailing theories about how periodontitis can influence pregnancy. The first postulates that the hematogenous dissemination of bacteria or their products through the bloodstream to the fetoplacental unit results in a localized infection and inflammatory response that alters the intrauterine immune status, leading to APOs.^16^ The second, more indirect theory suggests that bacterial products circulate through the bloodstream, resulting in a generalized systemic inflammatory response. Additionally, acute‐phase reactants from the maternal liver are thought to cross the placenta and reach the fetoplacental unit, altering the intrauterine immune status and causing APOs.^17^, ^18^

The latter is supported by findings showing that the number of *F. nucleatum* significantly increases during periodontitis in the gut, compared with healthy individuals,^19^, ^20^ suggesting that it is often swallowed. Their ability to form biofilms across diverse niches could enable them to colonize the gut.^7^ Alternatively, dysbiosis may result from metabolic endotoxemia arising from ingestion of live or dead Gram‐negative bacteria.^21^ Gut microbiome dysbiosis has also been associated with other disease states such as colorectal cancer, obesity, inflammatory bowel disease, ischemic heart disease, and possibly even mental disorders such as autism and depression.^22^, ^23^, ^24^, ^25^ *F. nucleatum*, in particular, has been shown to translocate to and colonize the gastrointestinal tract, where it promotes intestinal tumorigenesis and alters the tumor‐immune microenvironment.^26^, ^27^ Therefore, the present study aimed to determine whether periodontitis induced in mice by *F. nucleatum* and *P. gingivalis* would lead to changes in intestinal inflammation and the gut microbiome, and whether induced dysbiotic changes are associated with APOs. Our murine model was designed primarily to assess the impact of maternal periodontitis on gut inflammation and microbiota, with fetal and placental weights as proxy indicators of pregnancy health. While we discuss fetal growth restriction, placental dysfunction, and preterm delivery as relevant APOs in the human context, our endpoints were not intended or powered to detect those specific outcomes.

## 2 MATERIALS AND METHODS

### 2.1 Bacterial strains

*P. gingivalis* W50 (W83) and *F. nucleatum* (ATCC 10953) were cultured on anaerobic blood agar plates at 37°C under an anaerobic atmosphere of N_2_/CO_2_/H_2_ (90:5:5, v/v/v). After 72 h of anaerobic incubation on blood agar (Thermo Fisher, Waltham, MA, USA), 1.5 mL of 2% (w/v) carboxymethylcellulose (CMC, Sigma–Aldrich, St. Louis, MO, USA) was added to each plate, and bacterial cells were harvested directly using a sterile disposable spreader. The bacterial suspension was transferred to sterile 1.5 mL microcentrifuge tubes, and mice were inoculated within 30 min of harvesting. Bacterial density was estimated at ∼1 × 10^10^ CFUs/mL, corresponding to an optical density > 5.0 at 500 nm. The combined inoculum was prepared by mixing equal volumes (0.5 mL) of the *P. gingivalis* and *F. nucleatum* suspensions immediately prior to inoculation.

### 2.2 Murine periodontitis model

The murine periodontitis model used in this study is described by Stockham *et al.* ^28^ and subsequently used by multiple groups to induce reproducible periodontal inflammation and alveolar bone loss.^29^, ^30^, ^31^ Forty 8‐week‐old female BALB/c mice were acclimated for 1 week, then treated with 1 mg/mL kanamycin in drinking water for an additional week. Mice were randomly assigned to 2 groups: Group 1 (*n* = 20) received oral inoculation with *F. nucleatum* (ATCC 25586) and *P. gingivalis* (W50) suspended in 2% (v/v) CMC; Group 2 (*n* = 20) received 2% (v/v) CMC alone (vehicle control). Inoculations were administered daily for 5 weeks.^32^ Oral inoculations were performed by topically applying the bacterial suspension directly onto the gingival tissues using a sterile applicator. The inoculum was not delivered into the oropharynx and was not administered by gavage. Contamination was minimized by housing mice under specific‐pathogen‐free conditions in individually ventilated cages, with autoclaved chow and UV‐sterilized water provided ad libitum; bedding was changed twice weekly, and males were introduced only after completion of the inoculation period. Males were introduced simultaneously for 3 days. Male BALB/c mice were introduced at a 1:1 ratio for 3 days, and the presence of a vaginal plug marked the start of gestation, designated as gestational Day 1. All mice were housed at the University of Adelaide Animal Facility, maintained at 22 ± 2°C, 40%–60% humidity, on a 12‐h light/dark cycle (lights on at 7:00 a.m.). Bedding was changed twice weekly, and enrichment was provided. Mice were group‐housed (3–5 per cage), post‐mating. On gestational Day 18, mice were euthanized by CO_2_ asphyxiation, and tissues, including fetuses, placentas, gastrointestinal tract, major organs, parametrial fat, and blood, were collected. Skulls were prepared for microcomputed tomography (microCT) imaging to assess alveolar bone loss. All procedures were approved by the University of Adelaide Animal Ethics Committee (AEC M‐2015‐077) and complied with the National Health and Medical Research Council (NHMRC) Australian Code for the Care and Use of Animals for Scientific Purposes. Non‐pregnant cohorts were included as contextual controls to distinguish pregnancy‐specific effects from those attributable solely to periodontitis.

### 2.3 Assessment of alveolar bone loss

Following euthanasia, skulls were transferred to PBS and maintained hydrated at 4°C. Micro‐CT imaging was conducted within 48 h of tissue collection using a Skyscan 1076 high‐resolution desktop micro‐CT system (Bruker, Billerica, MA, USA) to minimize storage‐related artefacts and preserve native mineralized tissue morphology. Scans were performed at 60 kV and 170 µA using a 0.5 mm aluminum filter. Scans were carried out at 9 µm resolution, with a rotation step of 0.6° and frame averaging of 2. The maxillary molar region of each scan was reconstructed using NRECON version 1.6.6 (Bruker, Billerica, MA, USA), with a ring‐artifact reduction factor of 6 and a beam hardening correction rate of 30%. Images were then realigned using DataViewer version 1.5.2.4 (Bruker, Billerica, MA, USA). Reconstructed sagittal planes from each mouse were visualized in CTan version 1.18 (Bruker, Billerica, MA, USA). Alveolar bone loss was determined by measuring the distance between the alveolar bone crest (ABC) and the cemento‐enamel junction (CEJ) between the first and second maxillary molars at three planes and then averaged.

### 2.4 Measurement of serum cytokines

To assess systemic inflammatory mediator levels, blood samples collected on Day 18 were processed for serum isolation and then stored at −80°C until analysis. The presence of inflammatory mediators keratinocyte‐derived chemokine (KC), interleukin‐1β (IL)‐1, tumor necrosis factor‐α (TNF‐α), interleukin‐10 (IL‐10), interleukin‐6 (IL‐6), interleukin‐33 (IL‐33), receptor activator of nuclear kappa‐B ligand (RANKL), and lipopolysaccaride‐induced CXC chemokine (LIX) was detected using a multiplex assay. Additionally, C‐reactive protein (CRP) and serum amyloid A (SAA) were measured using ELISA. Upon thawing, serum was centrifuged at 13,000 × *g* for 10 min at room temperature, then diluted 1:20 (multiplex and SAA) or 1:10,000 (CRP), and assessed for cytokine levels according to the manufacturer's protocols (R&D Systems, Minneapolis, MN, USA). Multiplex assays were performed on a Luminex 200 System (Luminex Corporation, Austin, TX, USA), and concentrations were determined from standard curves generated with xPONENT software, version 3.1 (Luminex Corporation). ELISA assays were read at 450 nm using a PowerWave XS microplate reader and KC4 software to generate standard curves (BioTek Instruments, Winooski, VT, USA). All samples and standards were assayed in duplicate. The multiplex assay measured KC/CXCL1, IL‐1β, TNF‐α, IL‐10, IL‐6, IL‐33, RANKL, and LIX/CXCL5. Manufacturer‐specified lower limits of detection ranged from 1.0 to 4.6 pg/mL. CRP and SAA were quantified by ELISA with detection limits of 10 pg/mL and 1.0 ng/mL, respectively (R&D Systems, Minneapolis, MN, USA).

### 2.5 Histological analysis of gut sections

Histological examination of gut sections was performed to evaluate tissue inflammation in mice following periodontitis induction during pregnancy. All gastrointestinal tissues analyzed were collected from pregnant dams at gestational Day 18; fetal intestinal tissues were not examined. Sections from the jejunum and colon were collected in cassettes at post‐mortem and fixed in 10% formalin, followed by 70% ethanol. The sections were processed in a Leica TP1020 Tissue Processor (Leica Biosystems, Wetzlar, Germany) and embedded in paraffin wax. The embedded samples were then sectioned to a thickness of 5 µm using a microtome and stained with hematoxylin and eosin (H&E). Inflammation was scored via microscopy using critical indicators by 2 blinded researchers, as previously published.^33^ Where inflammatory scoring was discrepant, assessment was undertaken by a third researcher to reach consensus.

### 2.6 16S rRNA metagenomic gene sequencing

Metagenomic gene sequencing was conducted to determine the microbiological composition and abundance within the gut. Fresh fecal pellets were collected directly from individual mice by gentle abdominal massage into sterile, DNA‐free tubes, immediately snap‐frozen in liquid nitrogen, and stored at −80°C until DNA extraction. Bacterial genomic DNA was extracted from murine feces and caecum samples, using the QIAamp Fast DNA Tissue Kit (QIAGEN, Hilden, Germany) following the manufacturer's protocol. The extracted DNA was assessed for quantity and purity using a NanoDrop 2000c spectrophotometer (Thermo Fisher Scientific, Waltham, MA, USA) by measuring absorbance at 260/280 nm and the 260/230 nm ratio. Additionally, DNA integrity was verified via agarose gel electrophoresis to ensure the absence of degradation or contamination. Samples underwent Illumina library preparation using the Nextera XT DNA Library Prep Kit (Illumina, San Diego, CA, USA), which included enzymatic fragmentation, adapter ligation, and polymerase chain reaction (PCR) amplification. The V4 hypervariable region of the 16S rRNA gene was amplified using region‐specific primers with Illumina overhang adapters (515F/806R) to target conserved bacterial regions. Before sequencing, library concentrations were normalized using quantitative PCR (qPCR) and fluorometric quantification (Qubit dsDNA HS Assay, Thermo Fisher Scientific, Waltham, MA, USA). Amplicon sequencing was performed on the Illumina MiSeq platform (Illumina, San Diego, CA, USA) at Flinders Genomics Facility (Flinders University, South Australia), generating paired‐end reads (2 × 250 bp). The sequencing run included appropriate negative controls and mock bacterial communities to assess potential contamination and sequencing biases.

### 2.7 Processing raw 16S data

Raw sequences were processed using the microbiome‐dada2 pipeline (https://github.com/mucosal‐immunology‐lab/microbiome‐analysis/wiki/DataPreprocessing) using the DADA2 (version 1.28.0) R package.^34^ Briefly, indexed fastq files were demultiplexed using the iu‐demultiplex function (version 2.8) from illumina‐utils, primers and adapters removed with cutadapt (version 2.10), reads filtered and trimmed, sequencing error models generated, sequences dereplicated, amplicon sequence variants (ASVs) inferred, paired‐ends merged, and chimeras removed.^35^, ^36^ Bacterial 16S ASVs were assigned a taxonomy using the SILVA database train set (version 138.1) and the SILVA species assignment data set (version 138.1) for exact sequence matching. Samples with fewer than 5000 reads were excluded from the data set, and ASVs below 1% prevalence or unassigned at the Phylum level were filtered out. mbImpute (version 0.1.0) was applied to the 16S rRNA sequencing data to impute non‐biological zeros, using the sample group as a conditioning factor for imputation.^37^ Alpha diversity indices (Chao1, Simpson, and Shannon) were determined using the estimate richness function of the phyloseq (version 1.44.0) R package, and the Hill‐Shannon index was calculated using the hillR (version 0.5.2) R package.^38^, ^39^, ^40^ ASV counts were normalized by cumulative sum scaling using the calcNormFactors function from MetagenomeSeq (version 1.42.0), followed by log transformation. Statistical analyses were performed in R (version 4.3.0), and plots were generated using ggplot2 (version 3.4.2).^41^, ^42^, ^43^ Normality of data was determined using the Shapiro‐Wilk test, and subsequently, non‐parametric Wilcoxon Rank Sum tests assessed differences in bacterial alpha‐diversity using the rstatix (version 0.7.2) R package.^44^ Non‐parametric tests were used because the assumption of data normality was not upheld (significant variation from normality as assessed by Shapiro–Wilk normality tests). Principal coordinates analysis was performed on Bray‐Curtis distances using the ordinate function of the phyloseq package. A permutational multivariate analysis of variance (PERMANOVA) was performed using the adonis2 function from the vegan (version 2.6.4) R package to assess the effect of sample group on the ordination.^45^ Differential abundance testing for bacterial data was performed via a custom wrapper (see the differential abundance testing section of https://github.com/mucosal‐immunology‐lab/microbiome‐analysis/wiki/) around the R limma (version 3.56.0) function.^46^ A fixed random‐number seed value of 2 was set to ensure maximum reproducibility of tools requiring random pseudo‐numbers. A *p* value of less than 0.05 was considered statistically significant for all analyses. Multiple testing correction was not performed when running limma linear regression analyses to identify differentially abundant (DA) genera in the stool samples. Limma linear regression was used as a feature selection step, followed by post hoc Wilcoxon rank sum tests to determine significance.

### 2.8 Statistical analysis

The data were analyzed using a combination of GraphPad Prism version 10.2.0 (392) for Windows (GraphPad Software, CA, USA; www.graphpad.com), R (R Core Team), and IBM SPSS Statistics Version 24. Maternal data were corrected to reflect a viable litter size of 7.2. Unpaired, Welch‐corrected t‐tests were used to assess the statistical significance of maternal data. Multivariate sequencing data analysis was performed using PRIMER 7 with Permanova+ (PRIMER‐E Ltd., Auckland, New Zealand). The data were square‐root transformed and subjected to a Bray‐Curtis similarity matrix before PERMANOVA analysis. All experiments were performed in triplicate unless stated otherwise. Data plotted as mean ± SD, and a *p* value of <0.05 was deemed significant. Blinding was applied for outcomes involving subjective assessment (histological scoring), which was performed by two independent investigators, with discrepancies resolved by a third blinded assessor, whereas quantitative outcomes were analyzed using automated or objective readouts without investigator blinding.

## 3 RESULTS

### 3.1 Differences in pregnancy outcomes

**FIGURE 1:**
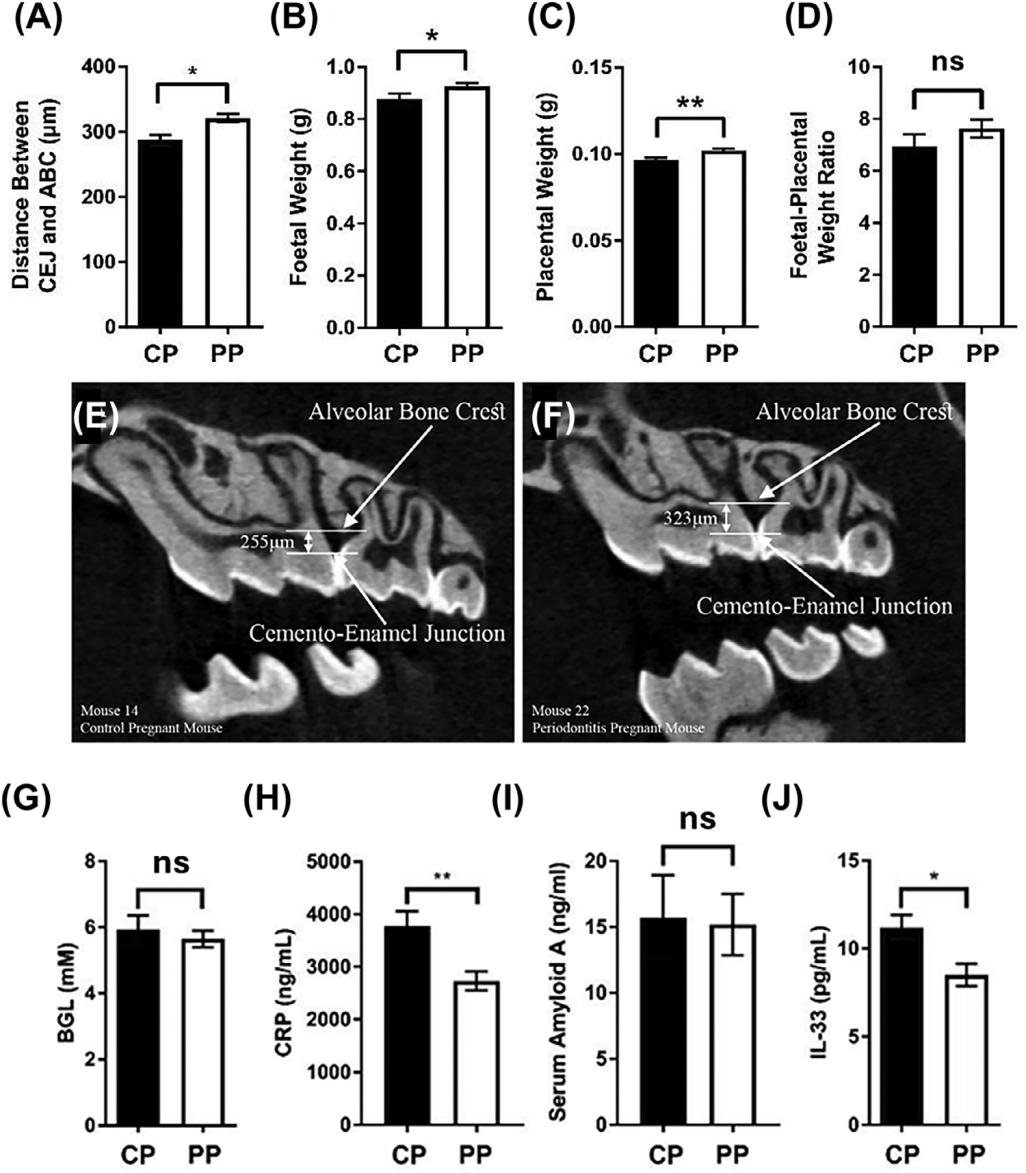
Effect of induced periodontitis on alveolar bone loss in pregnant mice and fetal and placental weight. (A) Mean distance between the cementoenamel junction and alveolar bone crest in CP mice (*n* = 9) and PP mice (*n *= 14;*p* = 0.018). Distances derived from measurements of Micro‐CT images. (B and C) Mean weight of fetuses and placentas from CP (*n *= 65) and PP (*n* = 100) mice (*p* = 0.0491 and *p* = 0.0025, respectively). (D) The ratio between fetal and placental weight for both CP (*n* = 65) and PP (*n* = 100) mice. Data presented as mean ± SD, * *p *< 0.05, ** *p *< 0.01, and ns represents non‐significant. (E) Micro‐CT image of the buccal view of maxillary left molars from a control mouse (CMC vehicle control) and (F) experimental mouse (*P. gingivalis* and *F. nucleatum* inoculation). Alveolar bone loss in (E) compared with (F) as indicated by the distance from the cemento‐enamel junction and alveolar crest. (G) Fasting blood glucose level (BGL) levels are measured after overnight fasting. (H) Serum CRP levels measured by ELISA. (I) Serum amyloid A concentration measured by ELISA. (J) Serum IL‐33 measured using Magnetic Luminex Screening Assay Data plotted as mean ± SEM; * *p* < 0.05; ** *p* < 0.01 and ns = non‐significant (*p* > 0.05).

Fourteen out of twenty experimental mice (*n* = 14) and 9 out of 20 control mice (n = 9) achieved pregnancy. Only pregnant mice were used for subsequent analysis. Data from non‐pregnant mice are presented for contextual comparison and are not a primary focus of the study. Previous studies have indicated that periodontal disease is a recognized risk factor for APOs.^15^, ^16^ The induction of periodontitis was confirmed by alveolar bone resorption in the treated groups. Pregnant periodontitis‐positive (PP) mice showed a significantly greater distance between the CEJ and ABC compared with pregnant control (CP) mice (*p* = 0.018) (Figure 1A,E and F), thus supporting attributions of further cohort differences to changes caused by periodontitis. In agreement with previous work,^28^ implantation sites and resorption rates were similar between the cohorts (data not shown), and the number of viable fetuses was also comparable, consistent with previous studies.^28^ Fetal weight increased by 5.74% ± 2.88%, and placental weight increased by 5.75% ± 1.86% in PP mice compared with their healthy counterparts (*p *< 0.05 and p < 0.01, respectively) (Figure 1B, C, and S1). However, these increased weights were not associated with an alteration in the fetal‐to‐placental weight ratio, which indicates a change in placental efficiency (Figure 1D).^47^ Additionally, examinations of the placental labyrinth and junctional zone/decidua showed no significant difference in the ratios between CP and PP mice.

### 3.2 Changes in systemic biomarkers

Analysis of systemic biomarkers revealed that the PP intervention selectively reduced inflammation without altering metabolic parameters. Blood glucose levels remained unchanged between the CP and PP groups, indicating that the intervention did not affect glycemic control (*p* = 0.133) (Figure 1G and Figure S2). Maternal organ weights, including the heart, lungs, spleen, kidneys, liver, and parametrial fat, also showed no significant differences between the CP and PP groups (*p* = 0.235, Figure S3). Induction of experimental periodontitis in PP mice significantly reduced CRP levels by 27.6% compared with CP mice (*p* = 0.003) (Figure 1H and Figure S4A). However, it is important to interpret this result within the context of species‐specific physiology. CRP is produced at relatively low levels in mice and exhibits a limited dynamic range in response to inflammatory stimuli, especially in chronic or localized conditions such as periodontitis.^48^ In contrast, no significant change in SAA concentrations was observed between the two groups (Figure 1I). IL‐33 concentrations were significantly lower in serum samples from PP mice (*p* = 0.043) (Figure 1J and Figure S4C).

### 3.3 Histological assessment of gastrointestinal inflammation

To evaluate whether periodontitis‐positive mice exhibited gastrointestinal inflammation, H&E stained sections of the jejunum and colon were examined. In the jejunum, inflammation scores were significantly elevated in the PP group compared with the CP group (2.5 ± 1.4; 0.2 ± 0.3, respectively; *p* < 0.001), indicating a robust inflammatory response in the small intestine (Figures S5A and S6A‐S6C). Similarly, the colon showed significantly higher inflammation scores in the PP group compared with the CP group (2.0 ± 1.3 and 0.8 ± 0.6, respectively; *p* < 0.05), although the effect was less pronounced than in the jejunum (Figures S5B and S6D).

### 3.4 Changes in gut microbiome composition—Alpha diversity is unaffected by periodontitis or pregnancy

**FIGURE 2:**
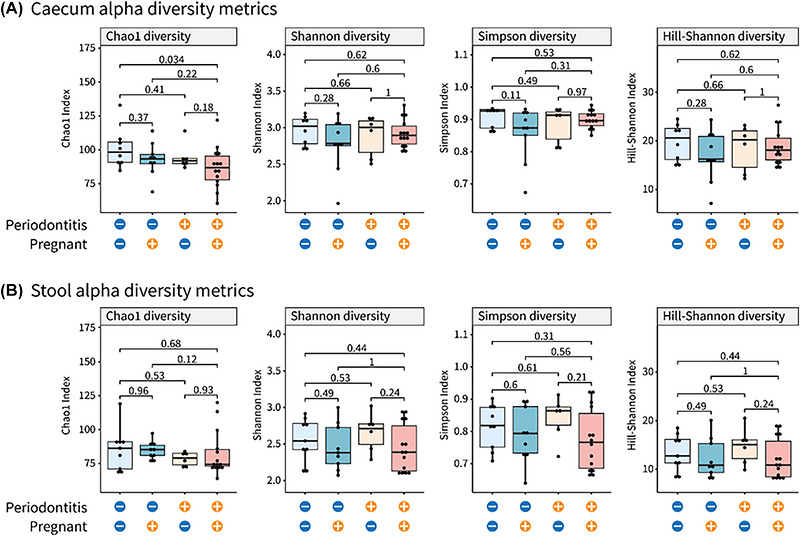
Periodontitis and pregnancy impact the alpha diversity metrics of the caecum and stool microbiota in a murine pregnancy model. (A) Boxplots representing bacterial alpha diversity metrics for caecum samples. (B) Corresponding boxplots for stool samples. Wilcoxon Rank Sum test results are shown.

Microbiome studies were conducted using samples from CP (*n *= 9) and PP (*n* = 14) mice. Bacterial alpha diversity appeared unaffected by periodontitis or pregnancy status and showed no differences between groups in caecum (Figure 2A) or stool samples (Figure 2B). The one exception was a modest reduction in absolute species richness (as measured by the Chao1 index) in periodontitis‐pregnant mice compared with control‐non‐pregnant mice (*p* = 0.03).

### 3.5 Bacterial beta‐diversity is unaffected by periodontitis or pregnancy

**FIGURE 3:**
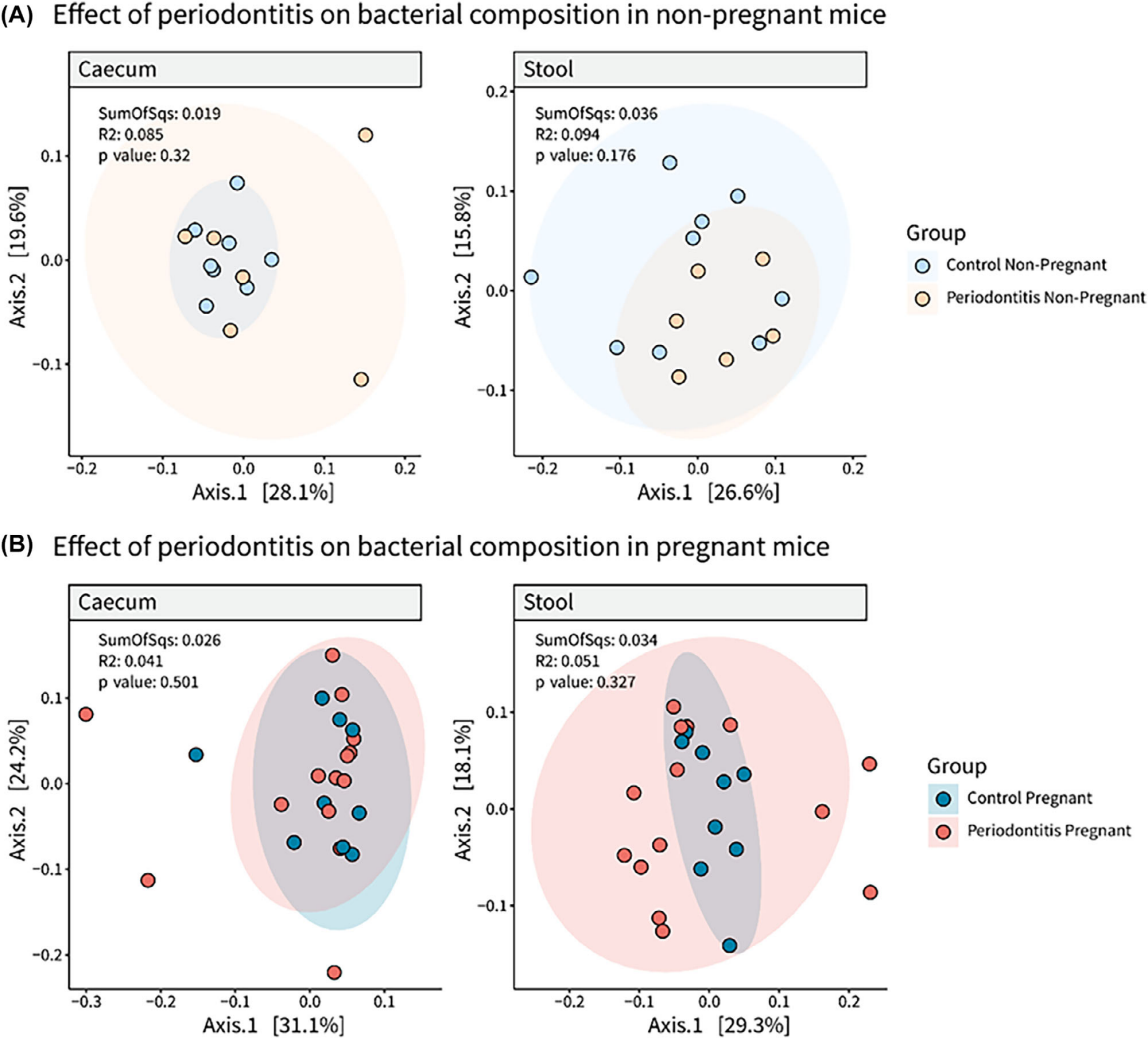
Effect of periodontitis on the gut microbiota composition in both non‐pregnant and pregnant mice. (A) PCoA ordination of Bray‐Curtis distances between samples from non‐pregnant mice. (B) Corresponding ordination plots for pregnant mice. Sample dots are colored by group, and the ellipse represents the 90% confidence interval for that group. PERMANOVA results are shown for the effect of periodontitis between groups (SumOfSqs is a measure of effect size and R2 indicates the variability explained).

To investigate the impact of periodontitis on gut microbial composition, Principal Coordinates Analysis (PCoA) was conducted using samples from both the caecum and stool of non‐pregnant (*n *= 9) and pregnant (*n *= 14) mice. In non‐pregnant mice (Figure 3A), microbial communities in the cecum and stool showed partial overlap between control and periodontitis groups, with slight shifts in clustering. However, two‐factor PERMANOVA analysis showed no significant difference in community composition (caecum: *R* ^2^ = 0.085, *p* = 0.32; stool: *R* ^2^ = 0.094, *p* = 0.176). This suggests that periodontitis induces only subtle alterations in gut microbiota structure, insufficient to reach statistical significance in the absence of pregnancy. In pregnant mice (Figure 3B), both caecal and stool samples showed high individual variation with greater dispersion in the periodontitis group. However, PERMANOVA again revealed no significant differences between groups (caecum: *R* ^2^ = 0.041, *p *= 0.501; stool: *R* ^2^ = 0.051, *p* = 0.327).

### 3.6 Pregnancy does not significantly alter gut microbial community structure in CP or PP mice

**FIGURE 4:**
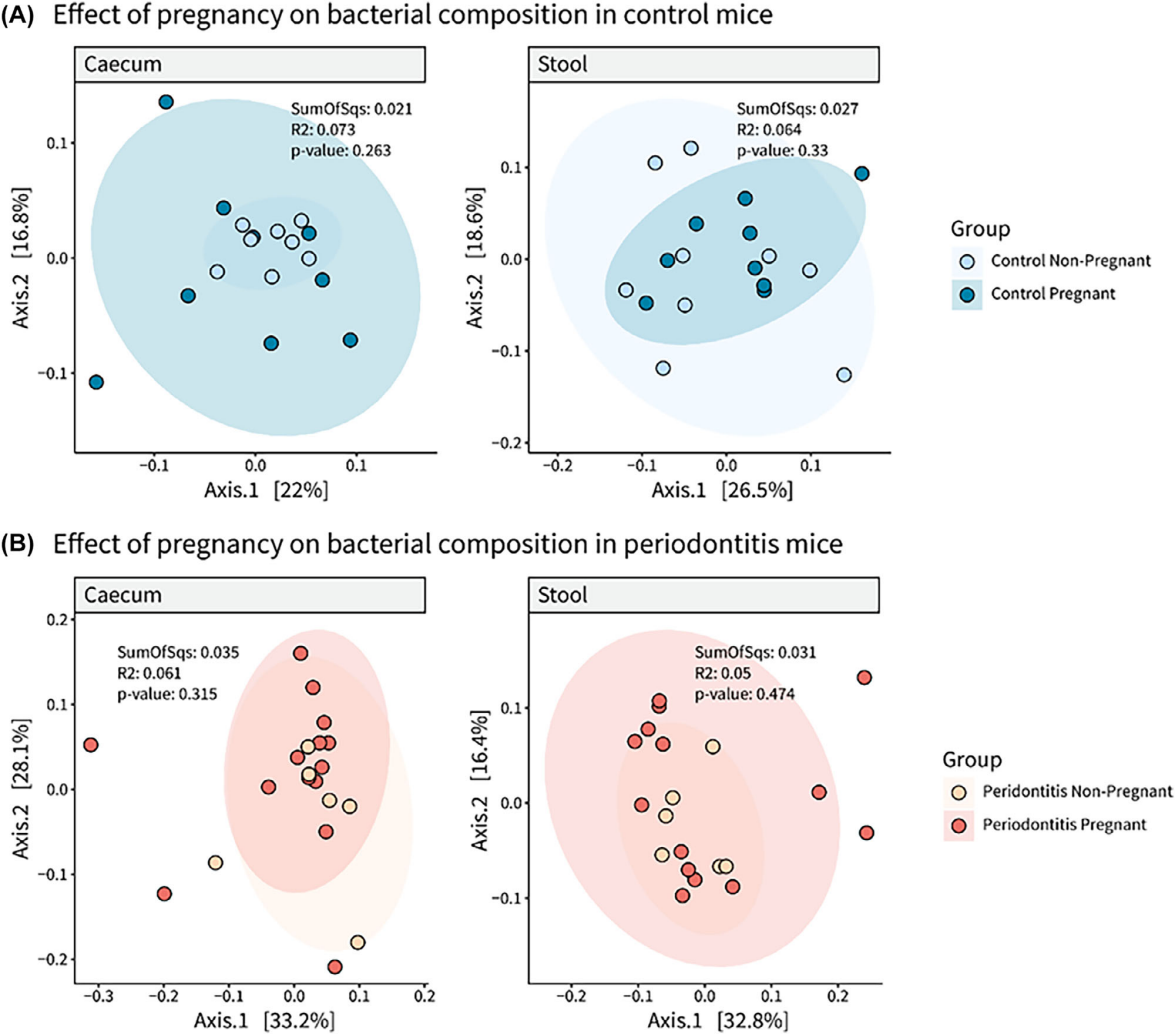
The impact of pregnancy on gut microbial community structure in control and periodontitis‐induced murine cohorts was evaluated by extended beta diversity analysis. (A) PCoA ordination of Bray–Curtis distances between samples from control mice. (B) Corresponding ordination plots for periodontitis mice. Sample dots are colored by group, and the ellipse represents the 90% confidence interval for that group. PERMANOVA results show the effect of pregnancy between groups (SumOfSqs is a measure of effect size, and R2 indicates the variability).

Figure 4 illustrates how pregnancy influences gut bacterial composition in both control and periodontitis‐induced mice, using Principal Coordinates Analysis (PCoA) of microbial profiles from the caecum and stool. In the control groups (Figure 4A), there is no significant difference in bacterial communities between non‐pregnant and pregnant groups (caecum: *R^2^* = 0.073, *p* = 0.263; stool: *R^2^* = 0.064, *p* = 0.33), suggesting pregnancy alone does not substantially alter gut microbiota in healthy mice. Similarly, in the context of periodontitis (Figure 4B), pregnancy does not significantly affect the gut microbiota in either the caecum (*R^2^* = 0.061, *p* = 0.315) or stool (*R^2^* = 0.050, *p* = 0.474). However, there appears to be increased dispersion in the pregnant periodontitis group, particularly in the caecal samples, suggesting elevated individual variability.

### 3.7 Taxonomic composition

**FIGURE 5:**
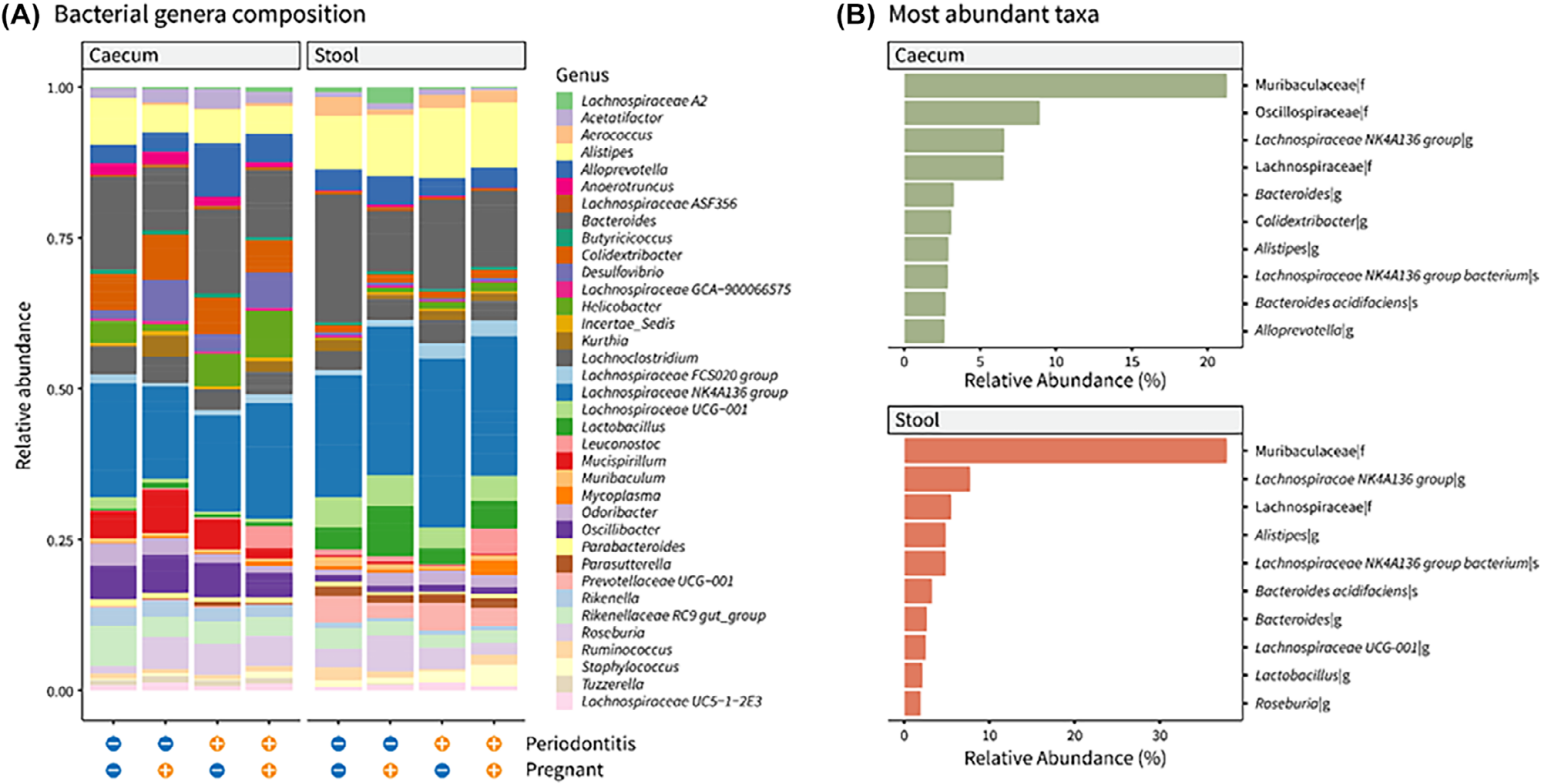
Summary of bacterial taxonomic composition of the gut microbiota across experimental groups. (A) Relative abundance of bacterial genera in both caecum and stool samples. A stacked bar plot representing periodontitis data, with pregnancy status indicated on the x‐axis. (B) Mean relative abundance for taxa at the deepest assignable level in both caecum and stool samples. Data are presented as percentages of the most abundant taxa.

Analysis of bacterial genera composition across caecum and stool samples revealed notable differences in microbial communities between the two intestinal niches (Figure 5A). The caecum exhibited a more heterogeneous bacterial profile, with notable representation of the *Lachnospiraceae*, *Oscillospiraceae*, and *Muribaculaceae* families. In contrast, stool samples showed a dominance of *Muribaculaceae*, followed by fewer and less diverse genera, suggesting compartment‐specific microbial adaptations. Despite compositional differences across anatomical locations, there were no consistent alterations in alpha diversity associated with either periodontitis or pregnancy status, as indicated by the stability of relative genus abundance across the experimental groups. As shown in Figure 5B, *Muribaculaceae* was the most abundant taxon in both caecal and stool samples, with relative abundance exceeding 20% in stool in the PP group. Other dominant taxa included *Oscillospiraceae*, *Lachnospiraceae NK4A136 group*, and *Bacteroides caecimuris*. These core microbial taxa persisted across all experimental groups regardless of pregnancy or periodontitis status, indicating a resilient gut microbiota structure that withstands systemic physiological changes. Importantly, taxa associated with short‐chain fatty acid production, such as *Lachnospiraceae* and *Ruminococcaceae*, remained stable.

### 3.8 Effect of periodontitis on the caecum microbiome

**FIGURE 6:**
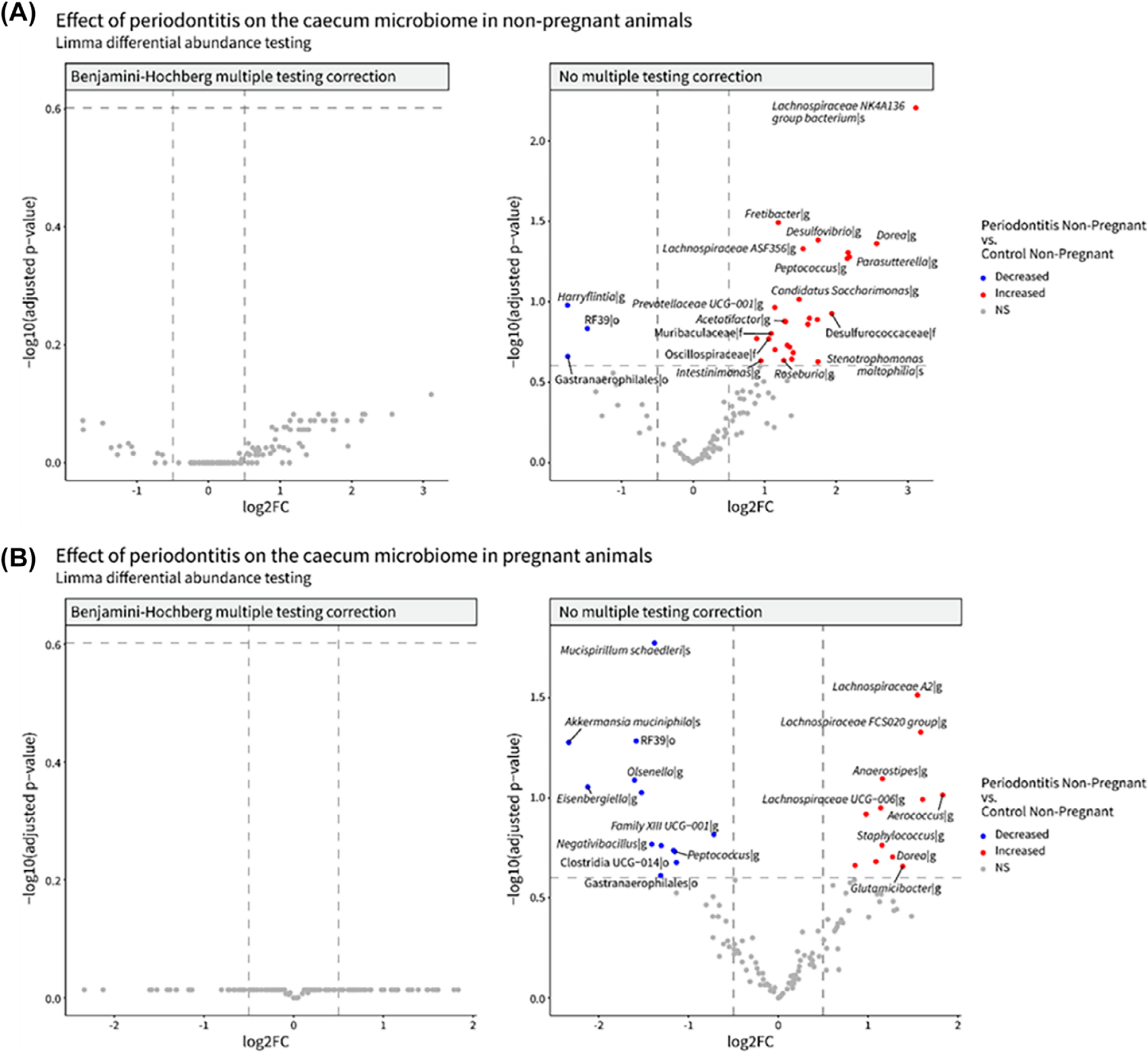
Volcano plots showing limma differential abundance testing (linear modeling) for caecum microbial taxa in (A) non‐pregnant mice, and (B) pregnant mice. Results are presented for differential abundance testing with and without Benjamini‐Hochberg multiple testing correction.

To further dissect the microbial shifts associated with periodontitis, we tested the caecal microbiota using a limma‐based linear modeling approach, with and without Benjamini‐Hochberg correction for multiple comparisons. In both non‐pregnant (Figure 6A) and pregnant cohorts (Figure 6B), no taxa reached statistical significance after adjustment for multiple testing, highlighting substantial inter‐individual variability in microbial profiles. However, exploratory analysis without correction revealed condition‐specific alterations in taxonomic abundance that may reflect biologically relevant trends. In non‐pregnant mice, periodontitis was associated with increased representation of genera such as *Lachnospiraceae NK4A136 group*, *Desulfovibrio*, and *Ruminococcus torques group*, alongside decreased abundance of *Muribaculaceae* and *Gastranaerophilales*. In pregnant mice, additional taxa appeared to be affected, with increases in *Lachnospiraceae FCS020 group*, *Aerococcus*, and *Streptococcus*, and significant reductions in *Akkermansia muciniphila* and *Mucispirillum schaedleri*.

### 3.9 Periodontitis and pregnancy alter the abundance of select immunomodulatory caecal bacteria

To investigate the taxa most influenced by periodontitis and pregnancy in the caecum, we evaluated the relative abundance (log‐transformed cumulative sum scaling‐normalized counts) of specific genera identified from differential abundance testing (Figure S7). *Akkermansia muciniphila* and *Mucispirillum schaedleri* abundance was significantly reduced in periodontitis pregnant mice (*p* < 0.05 and *p* < 0.001, respectively) compared with the non‐pregnant group. In contrast, *Desulfovibrio* was elevated considerably in the periodontitis pregnant group compared with the control non‐pregnant group (*p* < 0.01), highlighting a potential pro‐inflammatory microbial shift under dual exposure. *Lachnospiraceae FCS020* abundances were also significantly increased in periodontitis pregnant mice compared with the control non‐pregnant group (*p* < 0.05), suggesting synergistic effects of pregnancy and periodontal disease in enriching these taxa. In contrast, *Lachnospiraceae* NKA136, Oscillospiracaea, and *Peptococcus* group bacterium levels were elevated in periodontitis non‐pregnant mice (*p* < 0.05, *p* < 0.05, and *p* < 0.001, respectively), indicating a periodontitis‐specific effect independent of pregnancy.

### 3.10 Translocation of *P. gingivalis* and *F. nucleatum* to the placenta

We performed PCR analysis on placental tissue from murine pregnancies to assess whether the periodontitis‐associated oral pathogens *P. gingivalis* and *F. nucleatum* can disseminate from the oral cavity and translocate to the placenta in a mouse model. Interestingly, agarose gel electrophoresis of the PCR products revealed no detectable amplification of *P. gingivalis* or *F. nucleatum* DNA from placental tissue samples in our mouse model (Figure S8). This finding is notable, as other studies have reported translocation of these oral pathogens to the murine placenta following experimental infection.^49^, ^50^, ^51^

## 4 DISCUSSION

This study used a controlled murine model to investigate the impact of maternal periodontitis on gut microbiome composition and intestinal inflammation during pregnancy. Our findings provide compelling evidence that oral inflammation, while localized, can exert distal effects on gastrointestinal physiology and immune regulation, even in the absence of overt APOs. We interpreted these outcomes across key domains of analysis. The murine model effectively replicated periodontitis, as confirmed by alveolar bone loss detected by micro‐CT. While linear CEJ–ABC measurements are widely validated and reproducible for assessing periodontal bone loss in murine models, this approach does not capture full three‐dimensional or volumetric changes in alveolar bone, which should be addressed in future studies.

Interestingly, although the number of implantation sites and viable fetuses remained comparable between groups, periodontitis‐positive (PP) mice exhibited significantly higher fetal and placental weights. Increased fetal weight is commonly associated with maternal glucose intolerance, such as in cases of gestational diabetes.^52^ A previous study utilizing this murine periodontitis model found that mice with alveolar bone loss demonstrated significantly higher fetal weights and fetal/placental weight ratios without needing hematogenous translocation of *F. nucleatum* to the placenta.^28^ Additionally, adverse pathologies such as preterm birth and preeclampsia are reported in periodontitis cases and are connected to maternal glucose intolerance.^53^ Despite this, placental efficiency, as measured by the fetal‐to‐placental weight ratio, remained unchanged. These findings diverge from classical associations of periodontitis with fetal growth restriction or low birth weight and may reflect a pregnancy‐specific immune or metabolic compensation. One hypothesis is that periodontitis may subtly influence placental nutrient transfer without altering morphological zones such as the labyrinth. Alternatively, metabolic adaptations such as maternal glucose metabolism could underpin fetal overgrowth.^54^ Fasting blood glucose level (BGL) measurements taken at the time of post‐mortem were similar between both CP and PP mice. This contrasts with many observations made in human studies and previous animal models, in which periodontitis is linked to elevated BGL via its association with compromised glycemic control.^55^, ^56^ The overnight fasting protocol may have masked subtle glucose dysregulation, as recent studies recommend shorter fasting durations for optimal sensitivity in mice. Some studies suggest taking measurements after a 6‐h fasting period,^57^, ^58^ which may indicate that the overnight fasting period in our study was too long in a murine model. This period provides a superior index of glycemic control and has been shown to correlate more closely with glycated hemoglobin,^58^, ^59^ a more robust and integrative biomarker of long‐term glycemic control. Accordingly, the interpretation focuses on pregnancy‐specific outcomes, with non‐pregnant data serving primarily to contextualize these effects.

Contrary to the chronic inflammatory nature of periodontitis, systemic markers, including CRP and IL‐33, were significantly reduced in PP mice. While this may appear paradoxical, it aligns with the concept of pregnancy‐induced immune modulation,^60^ a protective mechanism that suppresses excessive systemic inflammation to maintain fetal tolerance. Notably, IL‐33 plays a dual role in promoting placental growth and exacerbating periodontitis by activating RANKL.^61^, ^62^ Its downregulation may indicate a rebalancing of immune priorities during gestation, with localized oral inflammation being immunologically compartmentalized. The concurrent reduction in IL‐33 and stable SAA levels suggests a selective systemic anti‐inflammatory profile, possibly evolved to prevent excessive immune activation that could compromise pregnancy outcomes.^63^ Together, these findings highlight the nuanced systemic immune adaptations during pregnancy, which may prevent the transduction of localized inflammation into systemic pathology, thereby preserving pregnancy outcomes despite ongoing oral disease. Histological assessment revealed significantly heightened inflammation in both the jejunum and colon of PP mice. The jejunum, in particular, exhibited a robust increase in inflammatory scores, indicating that the small intestinal mucosa is highly responsive to upstream oral inflammatory cues.^64^ This finding aligns with the emerging concept of bidirectional communication between oral inflammation and gastrointestinal inflammatory responses, whereby microbial translocation, swallowed oral pathogens, or circulating inflammatory mediators originating in the oral cavity may influence distal intestinal mucosal homeostasis.^65^ These intestinal changes occurred without widespread systemic inflammation, suggesting localized mucosal immune activation, potentially mediated by changes in gut microbial composition or direct pathogen‐associated molecular patterns that influence gut immune cells. These findings are critical, as they reveal that oral health disturbances can affect gut health independently of systemic cytokinemia, with implications for understanding inflammatory comorbidities during pregnancy. A limitation of this study is that periodontitis severity was assessed solely by alveolar bone loss in the maxillary molar region. While this site is widely used and sensitive for confirming disease induction in murine models, it does not capture disease extension across the entire dentition. Consequently, variability in overall periodontal burden may have influenced the limited systemic inflammatory response observed. Although the coexistence of local periodontal and intestinal inflammation with reduced systemic inflammatory markers, preserved gut microbiome structure, and increased fetal and placental weights appears to contrast with prior literature, pregnancy represents a unique immunological state characterized by immune tolerance and compartmentalization of inflammatory responses. Under these conditions, local inflammation may not translate into systemic cytokine elevation, and microbiome effects may be restricted to specific taxa rather than global diversity. Importantly, increased fetal and placental weights should not be interpreted as improved outcomes, but may reflect compensatory placental or metabolic adaptations.

Although the periodontitis model used in this study is based on oral colonization and confirmed alveolar bone loss, we acknowledge that a proportion of orally administered bacteria may be swallowed and contribute to gastrointestinal exposure. Importantly, this represents a biologically relevant mechanism rather than a methodological confound, as continual swallowing of oral pathobionts is an inherent feature of periodontitis in humans. Therefore, the observed intestinal inflammation and selective microbial shifts likely reflect the combined effects of periodontal inflammation–driven immune modulation and downstream oral–gut microbial transmission, consistent with the emerging concept of the oral–gut inflammatory crosstalk.

Consistent with the absence of overt APOs, placental PCR analysis did not detect *P. gingivalis* or *F. nucleatum* DNA in this model. While previous studies have reported placental colonization by oral pathobionts following experimental infection, such translocation appears to be highly model‐, dose‐, and timing‐dependent. The absence of detectable bacterial DNA in placental tissue here suggests that the observed intestinal inflammation and microbial shifts are unlikely to be driven by direct placental infection and instead supports indirect mechanisms such as immune modulation or oral–gut microbial transmission.

Although the study was designed to examine the established association between periodontitis and APOs, our findings indicate that the influence of periodontitis on the gut microbiota is most pronounced during pregnancy. Pregnancy is accompanied by profound hormonal, metabolic, and immunological adaptations that can alter mucosal immunity and host–microbiota interactions. These systemic changes may create a permissive environment in which periodontal inflammation exerts downstream effects on the gut microbiota that are not observed in the non‐pregnant state. Importantly, the absence of overt APOs suggests that these microbiome alterations occur within a framework of gestational immune tolerance and physiological compensation, rather than reflecting improved pregnancy health.

Microbiome profiling demonstrated remarkable stability in alpha‐ and beta‐diversity metrics despite histological inflammation and systemic perturbations. No significant differences in species richness or evenness were seen across pregnancy or periodontitis status, except for a slight reduction in Chao1 richness in PP mice. Similarly, beta diversity ordination revealed no statistically significant clustering of disease or gestational state. This microbial resilience may reflect the stability of core microbial taxa in the murine gut or limitations in sample size and taxonomic resolution. However, the increased dispersion observed in PP mice, particularly in caecal samples, suggests greater inter‐individual variability, a hallmark of stress‐related dysbiosis. These findings align with previous reports that pregnancy‐associated microbial shifts may be more subtle or site‐specific and often require high‐resolution or functional profiling to detect.^66^, ^67^, ^68^

Despite the lack of broad diversity changes, taxonomic analyses identified subtle yet potentially significant alterations in the gut microbiota. In particular, PP mice exhibited reductions in beneficial taxa, such as *Akkermansia muciniphila* and *Mucispirillum schaedleri*, which are implicated in maintaining mucosal integrity and regulating the immune system. This reduction is particularly noteworthy, as *Akkermansia* plays a key role in maintaining gut barrier integrity and exerting anti‐inflammatory effects,^69^, ^70^ while *Mucispirillum schaedleri*, a mucosal colonizer, may contribute to immune regulation.^71^ Conversely, an increase in pro‐inflammatory taxa, such as *Desulfovibrio* and the *Lachnospiraceae* FCS020 group, was observed. These shifts suggest a microenvironment that may favor low‐grade mucosal inflammation or compromised barrier function, consistent with the histological findings. The enrichment of sulfate‐reducing bacteria, such as Desulfovibrio, is particularly noteworthy, given their association with hydrogen sulfide production and intestinal inflammation.^72^ These findings suggest that pregnancy and periodontitis may exacerbate compositional shifts in caecal microbiota, particularly impacting immunomodulatory and mucus‐associated genera. Although these results should be interpreted cautiously given the lack of statistical correction, they align with previously reported studies.^73^, ^74^, ^75^ Taxa‐level perturbations warrant further targeted validation in future studies.

Several limitations of this study warrant consideration. Although swallowed oral bacteria may contribute to gastrointestinal microbial exposure, the presence of statistically significant alveolar bone loss confirms that the observed intestinal and immune effects occurred in the context of established periodontal disease rather than nonspecific bacterial gavage. While the murine model allows for experimental control, its relevance to human pregnancy remains limited, and caution is therefore required when extrapolating these findings to clinical settings. In addition, while fetal and placental weights, placental efficiency, and placental zone proportions were assessed, the study was not designed to comprehensively profile fetoplacental inflammatory biomarkers or perform microbiome sequencing of these low‐biomass tissues. Such analyses would require dedicated experimental design, extensive contamination controls, and additional powering and were therefore beyond the scope of the present study. Additionally, the relatively small sample size and cross‐sectional design may have limited our ability to detect subtle or dynamic changes in microbial communities over time. Furthermore, reliance on 16S rRNA gene sequencing restricted taxonomic resolution and precluded functional inference, highlighting the need for future studies employing longitudinal designs and multi‐omics approaches.

While this study was conducted in a murine model, the findings raise important questions about the translational potential to human pregnancy. Given the observed impact of periodontitis on intestinal inflammation and microbial composition, routine dental screening and periodontal care could represent a low‐risk, preventative strategy to support maternal gut health during pregnancy. Integrating oral health assessments into prenatal care may help mitigate the systemic inflammatory burden and improve long‐term outcomes for both the mother and the child.

## 5 CONCLUSIONS

This study shows that periodontitis during pregnancy is associated with small intestinal inflammation and selective changes in gut microbial composition, including reduced *Akkermansia muciniphila* and increased *Desulfovibrio*, without overt impairment of core pregnancy outcomes in mice. The accompanying reductions in CRP and IL‐33 suggest systemic immune modulation, reinforcing the existence of oral–gut inflammatory crosstalk during gestation. While adverse fetal effects were not observed, the presence of gut dysbiosis and inflammation highlights the broader systemic impact of oral disease in pregnancy. These findings underscore the importance of managing maternal periodontal health as a potential avenue to safeguard gastrointestinal and immune homeostasis, with implications for maternal and offspring well‐being.

## AUTHOR CONTRIBUTIONS

**Richard Bright**: Conceptualization; methodology; formal analysis; data curation; writing—original draft; writing—review & editing. **Matthew G. Macowan**: Investigation; data curation; formal analysis; writing—review & editing. **Keyuan Tian**: Investigation; data curation; formal analysis; writing—review & editing. **Tracy Fitzsimmons**: Investigation; data curation; writing—review & editing. **Rebecca L. Wilson**: Investigation; data curation; formal analysis; writing—review & editing. **Claire T. Roberts**: Formal analysis; writing—review & editing. **Claus T. Christophersen**: Formal analysis; data analysis (microbiome bioinformatics); writing—review & editing. **Peter M. Bartold**: Conceptualization; supervision; writing—review & editing. **Stephen P. Kidd**: Conceptualization; supervision; writing—review & editing. **Peter S. Zilm**: Conceptualization; methodology; supervision; funding acquisition; writing—review & editing.

## CONFLICT OF INTEREST STATEMENT

The authors declare that they have no known competing financial interests or personal relationships that could have influenced the work reported in this paper.

## Supporting information



Supporting information

## Data Availability

The data supporting this study's findings are available from the corresponding author upon reasonable request.
