# Supplementary material for: Periodontitis during pregnancy: The effect on the gut microbiome and intestinal inflammation
Source: J Periodontol. 2026 Apr 14;97(7):1587–603. doi: 10.1002/jper.70132 (PMC13380379; doi:10.1002/jper.70132)
Supplement: Supplementary file 1 — Supporting information [file JPER-97-1587-s001.docx]

**Supplementary Material**

**Periodontitis During Pregnancy: the effect on the gut microbiome and Intestinal Inflammation**

Richard Bright^1*^, Matthew G. Macowan^2^, Keyuan Tian^3^, Tracy Fitzsimmons^3^, Rebecca L. Wilson^4^, Claire T. Roberts^1^, Claus T. Christophersen^5^, Peter M. Bartold^3^, Stephen P. Kidd^2^, and Peter S. Zilm^3*^

^1^College of Medicine and Public Health, Flinders University, Bedford Park, South Australia, 5042, Australia

^2^School of Biological Sciences, University of Adelaide, SA, 5005, Australia

^3^Adelaide Dental School, University of Adelaide, SA, 5005, Australia

^4^Robinson Research Institute, University of Adelaide, SA, 5005, Australia

^5^School of Medical and Health Sciences, Edith Cowan University, WA, 6027, Australia

**Corresponding authors:** [richard.bright@flinders.edu.au](mailto:richard.bright@flinders.edu.au) and [peter.zilm@adelaide.edu.au](mailto:peter.zilm@adelaide.edu.au)

**Keywords:** Periodontitis, 16S rRNA sequencing, oral–gut axis, gut microbiome, microbiota dysbiosis, inflammation


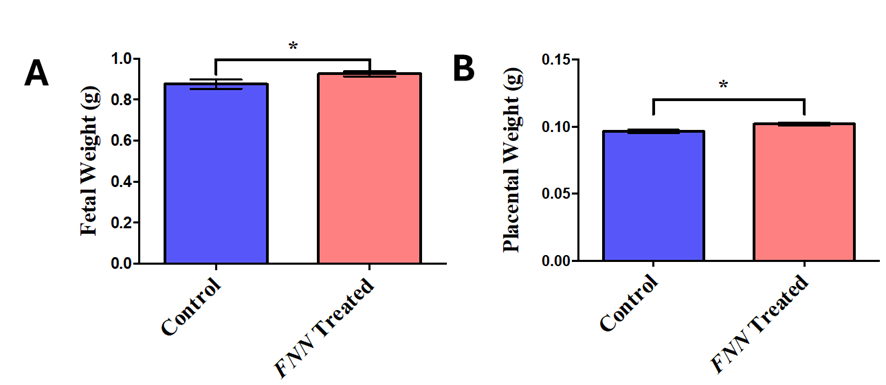


**Figure S1**. Effect of *P. gingivalis* and FNN-induced periodontitis on (**A**) fetal and (**B**) placental weights. Data plotted as means ± SEM, *n* = 6 and * *p* < 0.05.


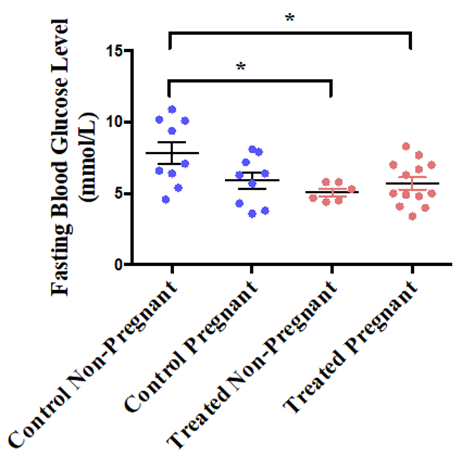


**Figure S2.** Fasting blood glucose (mmol/L) was measured in control and treated pregnant and non-pregnant animals. Data are shown as mean ± SEM (n = 10–13 per group) and * p <0.05.


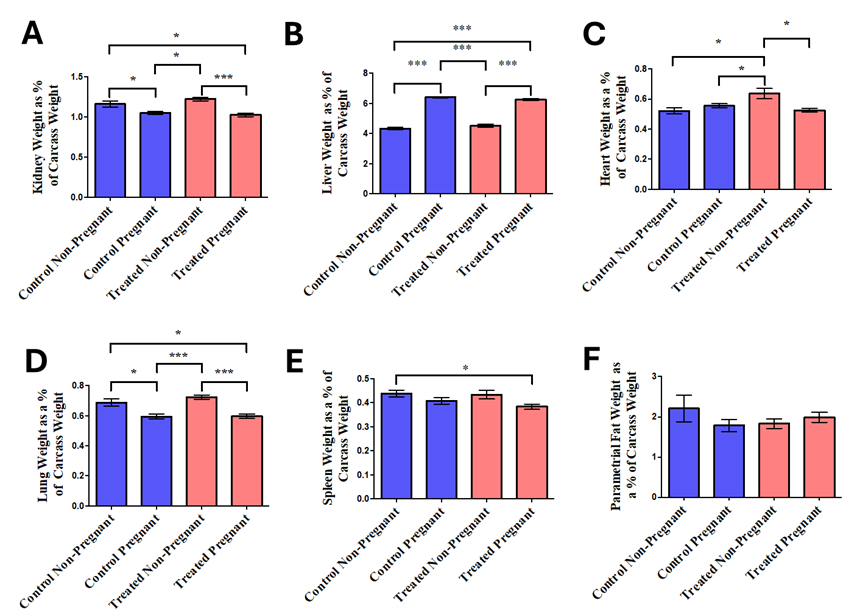


**Figure S3**. Organ weights as a percentage of carcass weight across experimental groups. Relative organ weights were assessed in control and treated animals, both non-pregnant and pregnant. for (**A**) kidneys, (**B**) liver, (**C**) heart, (**D**) lungs, (**E**) spleen, and (**F**) parametrial fat. Treatment significantly reduced liver and kidney weights and modulated heart and lung weights across groups. Data Plotted as mean ± SEM, *n* =3, **p* < 0.05, ***p* < 0.01, ****p* < 0.001, *****p* < 0.0001.


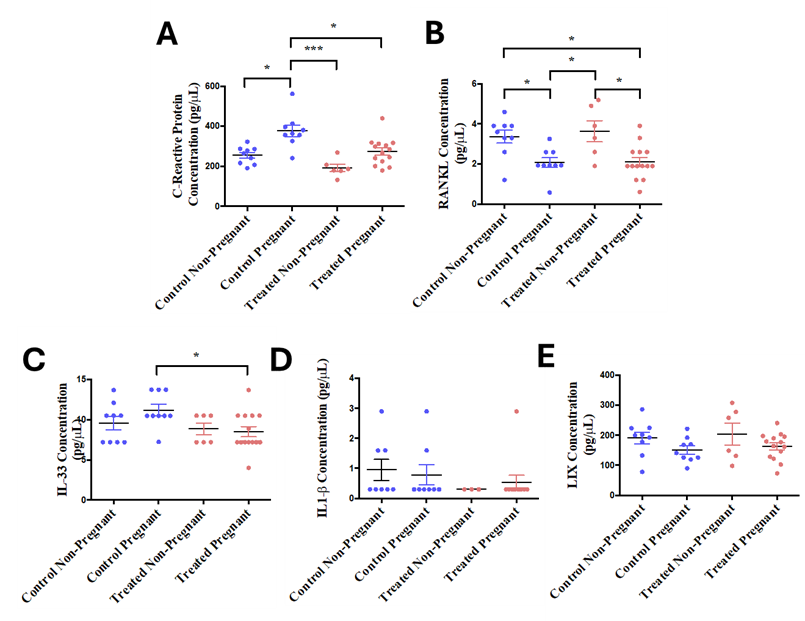


**Figure S4**. Circulating inflammatory biomarker levels across experimental groups. Serum concentrations of inflammatory markers were measured in control and treated animals, both pregnant and non-pregnant. (**A**) C-reactive protein (CRP), (**B**) RANKL, (**C**) IL-33, (**D**) IL-1β, and **(E**) LIX. Data are presented as mean ± SEM (*n* = 7–16 per group); **p* < 0.05 and ***p < 0.001.


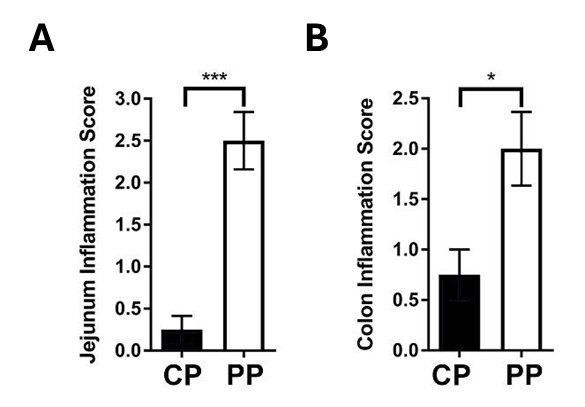


**Figure S5**. Induction of periodontitis increases gastrointestinal inflammation in pregnant mice. Inflammation score of (**A**) jejunum samples and (**B**) colon samples from a random selection of CP mice. Two independent assessors scored each sample, and the values were combined by cohort. Data plotted as mean ± SD, (CP) *n* = 9, (PP) *n* = 14, **p* < 0.05 and *** *p* < 0.001.


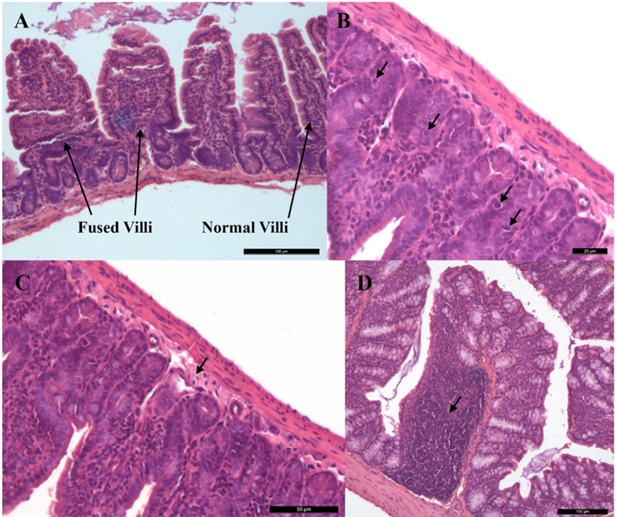


**Figure S6**. Representative hematoxylin and eosin (H&E)–stained sections of the jejunum and colon illustrating inflammatory and structural changes. (A) Jejunum from control pregnant (CP) mice showing fused villi (scale bar = 100 µm). (B) Jejunum from periodontitis-pregnant (PP) mice showing crypt disruption (arrows), appearing as dark-stained regions surrounded by a pale halo (scale bar = 20 µm). (C) Jejunum from PP mice showing mild oedema, with separation of the muscularis mucosae from the base of the crypts and evidence of fluid infiltration (scale bar = 50 µm). (D) Colon section showing a prominent lymphoid aggregate (arrow) (scale bar = 100 µm).


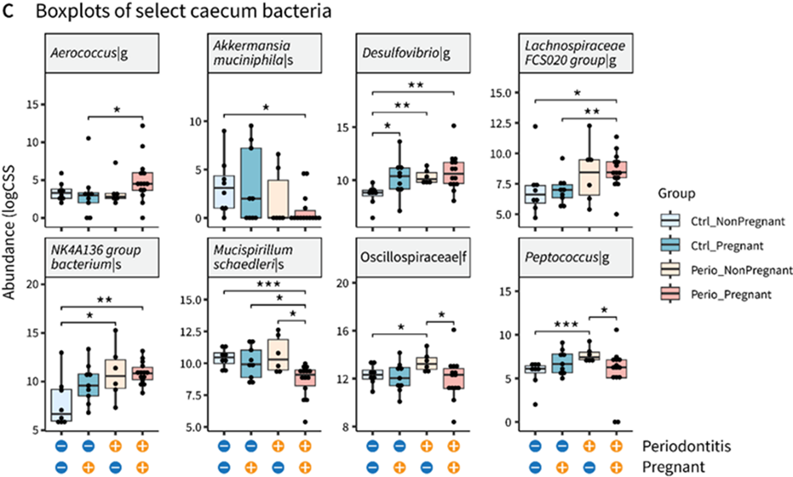


**Figure S7.** Select caecum bacterial taxa affected by periodontitis and pregnancy. Symbols indicate statistical significance from pairwise Wilcoxon tests: **p* < 0.05, ** *p* < 0.01, *** *p* < 0.001 and *n* =3.

**Detection of *P. gingivalis* and *F. nucleatum* in placental tissue by polymerase chain reaction (PCR)**

DNA Extraction

Placental tissue samples were aseptically collected and stored at –80°C until processing. Genomic DNA was extracted using the QIAwave DNA Blood and Tissue Kit (Qiagen, Hilden, Germany) according to the manufacturer’s protocol, with slight modifications to enhance bacterial DNA recovery. Approximately 500 mg of placental tissue was briefly homogenized in lysis buffer with Proteinase K and incubated at 56°C until complete digestion. DNA was then eluted in 50 µL of elution buffer and quantified using a NanoDrop spectrophotometer (Thermo Fisher Scientific, MA, USA).

**PCR amplification**

Polymerase chain reaction was employed to detect the presence of *P. gingivalis* and *F. nucleatum* using species-specific primers targeting the 16S rRNA gene. The sequences of the primers were as follows:

*P. gingivalis*:

Forward: 5′-AGGCAGCTTGCCATACTGCG-3′

Reverse: 5′-ACTGTTAGCAACTACCGATGT-3′

*F. nucleatum*:

Forward: 5′-CGCAGAAGGTGAAAGTCCTGTAT-3′

Reverse: 5′-TGATGACGTTACCCCGCAGAA-3′

PCR reactions were performed in 25 µL volumes containing 1× PCR buffer, 2.5 mM MgCl₂, 0.2 mM dNTPs, 0.5 µM of each primer, 1 U of Taq DNA polymerase, and 100 ng of template DNA. The thermocycling conditions were as follows: initial denaturation at 95°C for 5 minutes; 35 cycles of denaturation at 95°C for 30 seconds, annealing at 60°C for 30 seconds, and extension at 72°C for 45 seconds; followed by a final extension at 72°C for 5 minutes.

**Agarose gel electrophoresis**

PCR products were resolved on a 1.5% agarose gel containing ethidium bromide (0.5 µg/mL) in Tris-acetate-EDTA buffer. Electrophoresis was conducted at 100 V for 45 minutes, and bands were visualized under UV illumination using a gel documentation system (ThermoFisher, MA, USA). Positive detection of *P. gingivalis* and *F. nucleatum* was determined by the presence of amplicons at the expected sizes (404 bp and 360 bp, respectively).


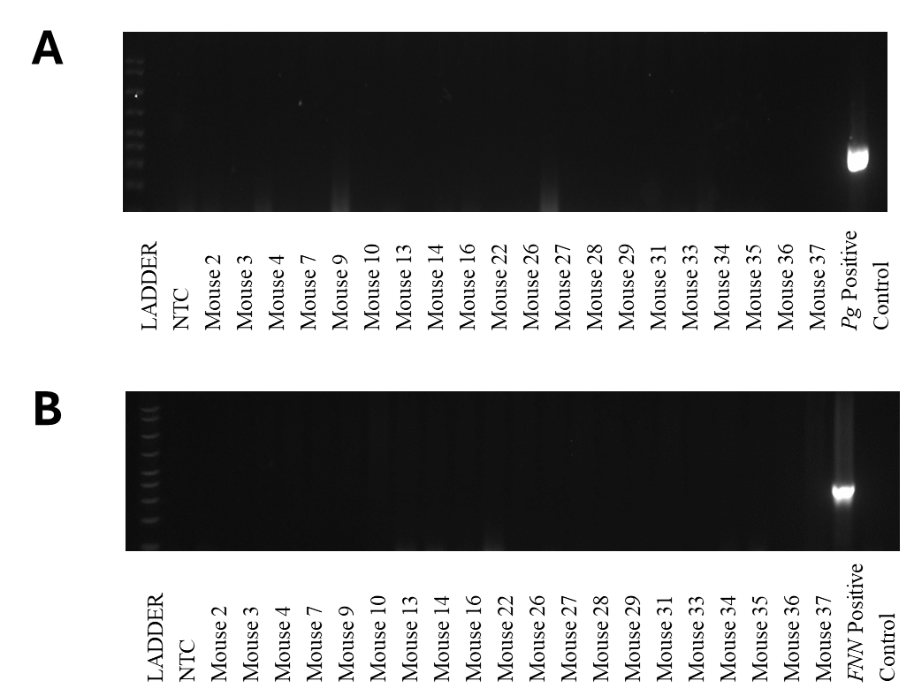


**Figure S8.** Agarose gel of PCR amplicons from (A) *P. gingivalis* and (B) FNN-specific primers in placental tissue.
